# Supplementary material for: Dissecting the Nuclear Import of the Ribosomal Protein Rps2 (uS5)
Source: Biomolecules. 2023 Jul 14;13(7):1127. doi: 10.3390/biom13071127 (PMC10377357; doi:10.3390/biom13071127)
Supplement: Supplementary file 1 [file biomolecules-13-01127-s001.zip › SupplementaryData.pdf]

SUPPLEMENTARY DATA

Dissecting the nuclear import of the ribosomal protein Rps2 (uS5)

Andreas Steiner [1,2], Sébastien Favre [3], Maximilian Mack [1,2], Annika Hausharter [1], Benjamin Pillet [3], Jutta Hafner [1,2], Valentin Mitterer [1,2], Dieter Kressler [3], Brigitte Pertschy [1,2,*] and Ingrid Zierler [1,2,*]

[1] Institute of Molecular Biosciences, University of Graz, Humboldtstrasse 50, 8010 Graz, Austria
[2] BioTechMed-Graz, Mozartgasse 12/II, 8010 Graz, Austria
[3] Unit of Biochemistry, Department of Biology, University of Fribourg, Chemin du Musée 10, 1700 Fribourg, Switzerland
* Email of the corresponding authors: brigitte.pertschy@uni-graz.at; ingrid.zierler@uni-graz.at.

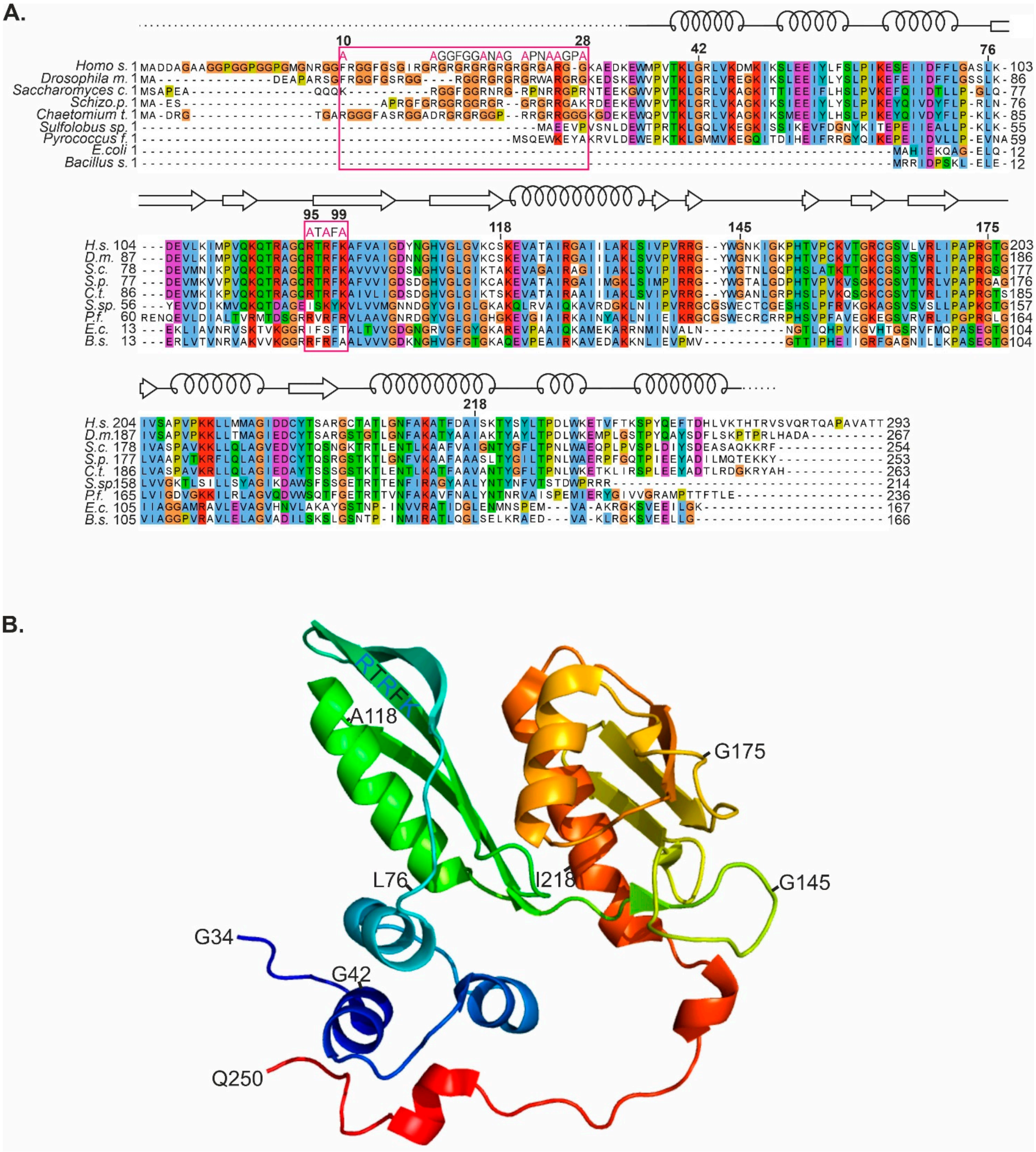

Supplementary Figure S1. **Sequence and structure of Rps2. A.** Sequence alignment of the highly conserved r-protein Rps2. Sequences were aligned with Clustal Omega and viewed in Jalview. Mutations and fragments of Rps2 used in this study are indicated. **B.** Structure of Rps2 (from PDB 4V88, [44]) in rainbow color representation with borders of the fragments used in this study, as well as the sequence ranging from amino acids 95 to 99 of Rps2 (RTRFK) indicated.

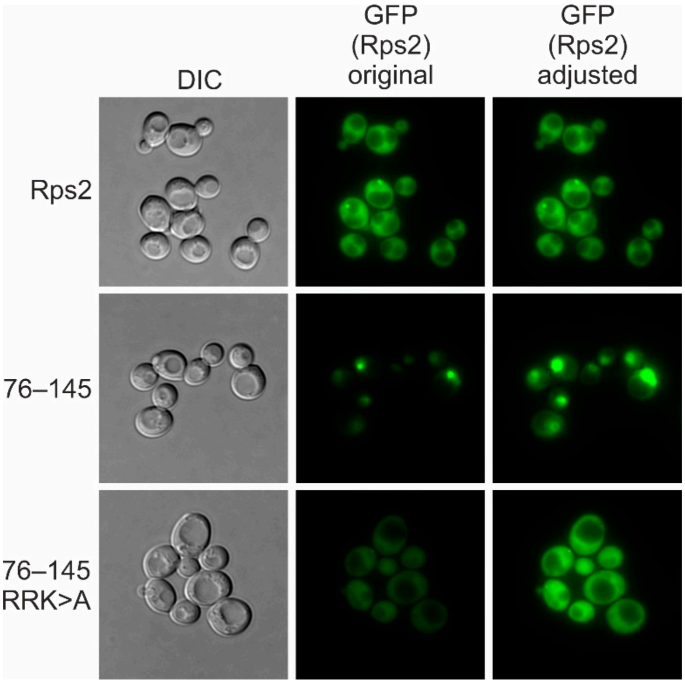

Supplementary Figure S2. **Localization of Rps2(76–145).$R_{95}R_{97}K_{99}$>A-3xyEGFP.** The images for which the intensities were adjusted in Figure 2B are shown in both the adjusted and the original, identically processed version.

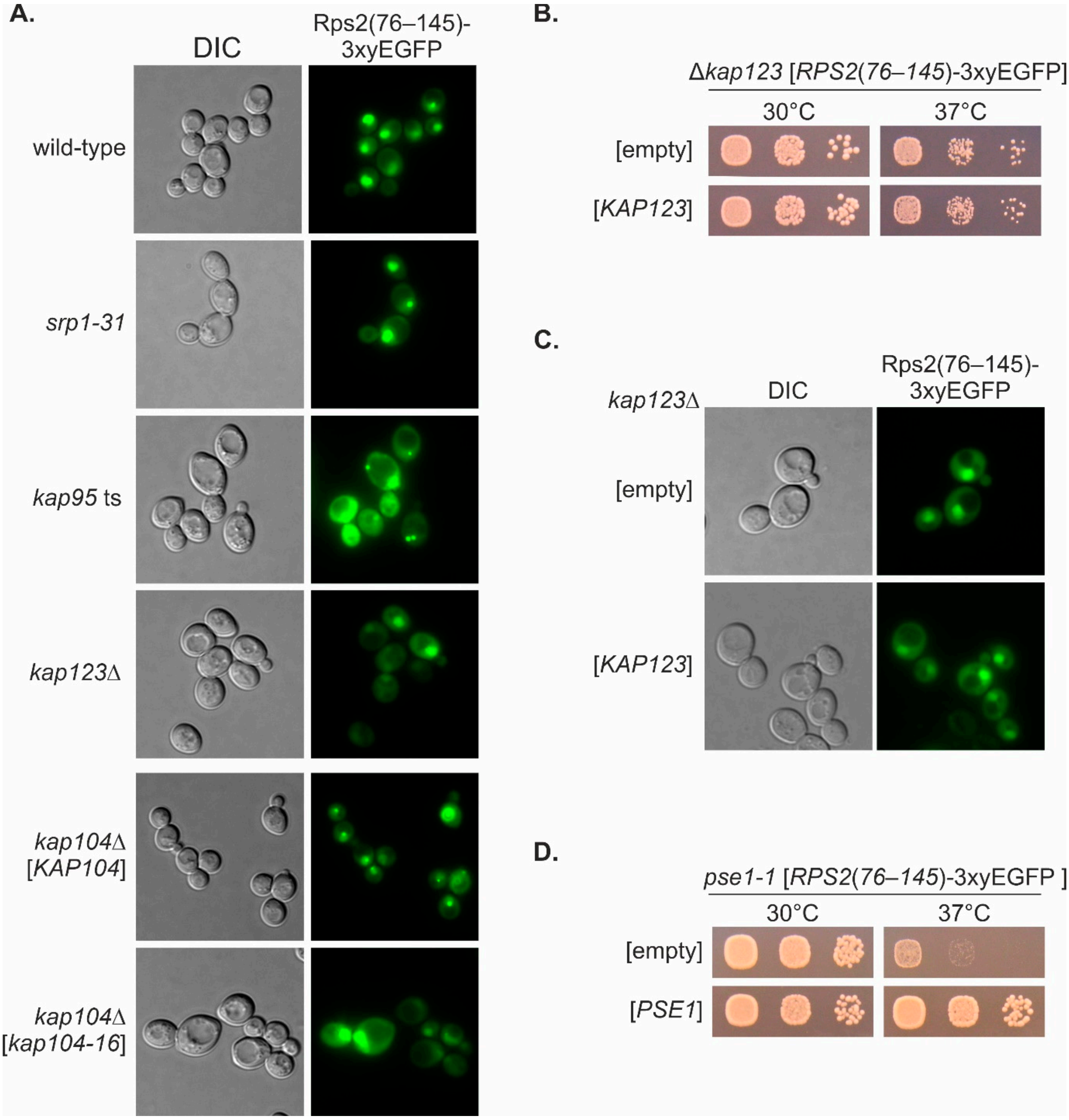

Supplementary Figure S3. **Nuclear import of Rps2(76-145)-3xyEGFP. A.** Localization of Rps2(76–145)-3xyEGFP in the wild-type strain, the importin mutant strains *srp1-31, kap95* ts, *kap123*Δ, and the *kap104*Δ strain either transformed with a plasmid carrying *KAP104* (control) or the mutant *kap104-16* allele. **B.** Complementation assay. The *kap123*Δ strain was transformed with a *KAP123*-harboring *TRP1* plasmid or the empty control plasmid, as well as with the Rps2(76–145)-3xyEGFP *LEU2* reporter plasmid. Transformed cells were spotted in 10-fold serial dilution steps onto SDC-leu-trp plates, which were incubated at 30°C or 37°C for three days. **C.** Transformants from B were inspected by fluorescence microscopy. **D.** The *pse1-1* transformants analyzed in Figure 3B were spotted in 10-fold serial dilution steps onto SDC-leu-ura plates, which were incubated at 30°C or 37°C for three days.

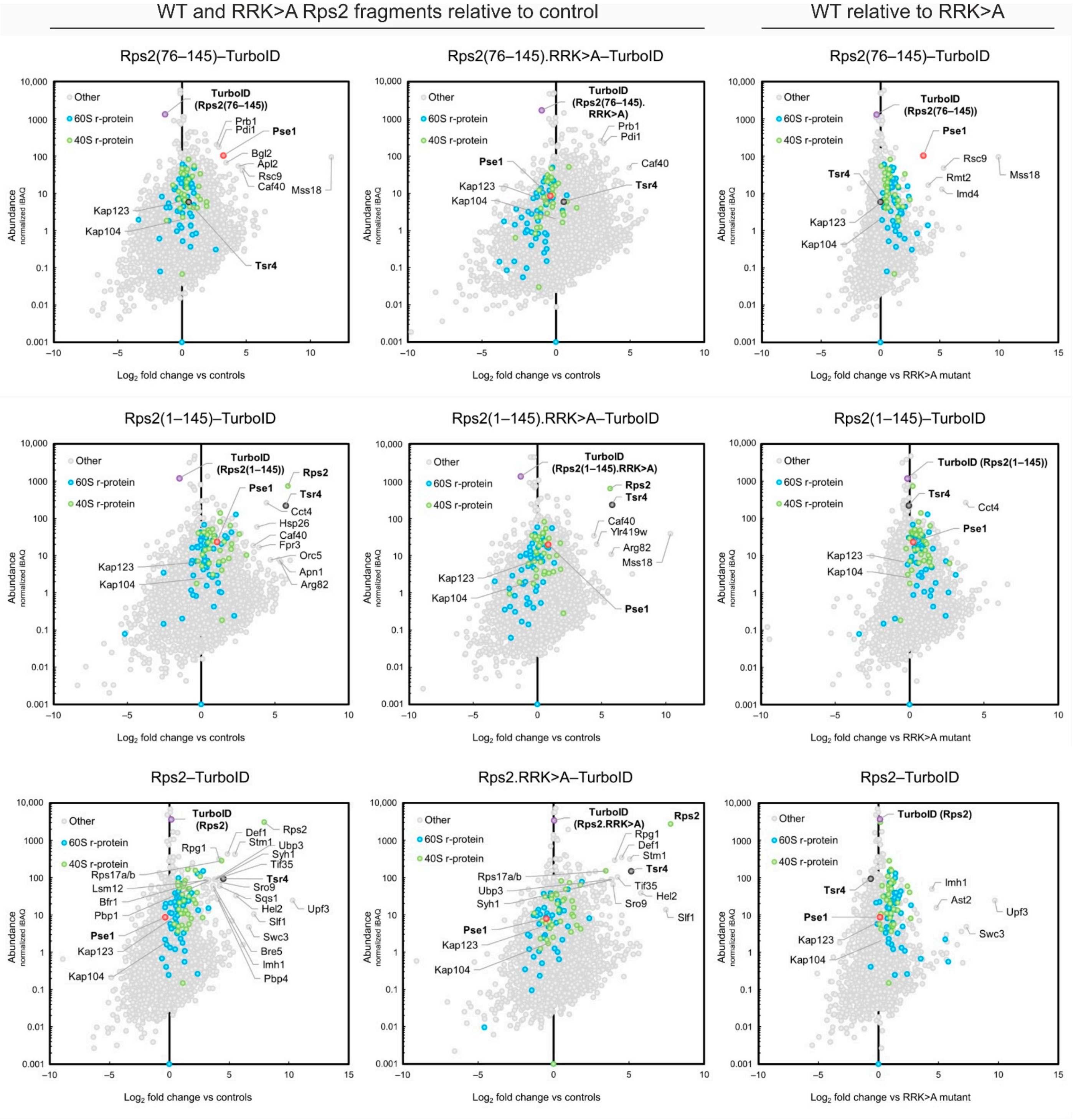

Supplementary Figure S4. **TurboID-based proximity labeling using Rps2, Rps2(1–145), and Rps2(76–145), all with and without the $R_{95}R_{97}K_{99}$>A exchanges, as baits.** The normalized abundance value (iBAQ) of each protein detected in the respective purification is plotted against its relative abundance (log_2-transformed enrichment) compared to the abundance in control or related purifications. Relative abundance was either calculated compared to the averaged protein abundance in the two control purifications (left two panels; derived from cells individually expressing the GFP-TurboID and the NLS-GFP-TurboID bait, which accounts for the cytoplasmic and nuclear background, respectively) or the respective Rps2 wild-type variant was compared to the same TurboID-tagged bait containing the $R_{95}R_{97}K_{99}$>A exchanges. The names of proteins that are particularly enriched, as well as importins Pse1, Kap123 and Pse1 are indicated. The bait proteins, Tsr4, and Pse1 are highlighted by bold letters.

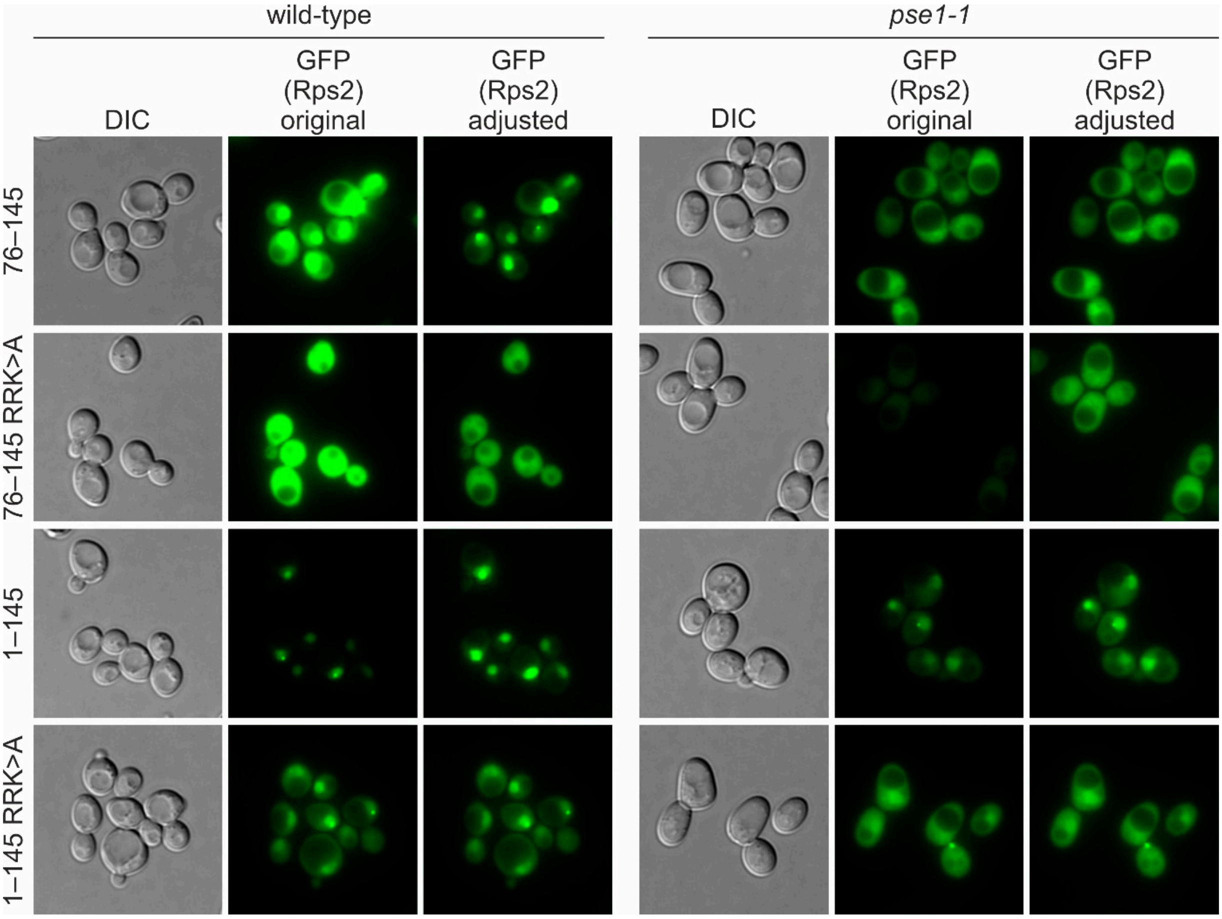

Supplementary Figure S5. **Localization of Rps2-3xyEGFP variants in the wild-type and *pse1-1* mutant strain.** The images for which the intensities were adjusted in Figure 4A are shown in both the adjusted and the original, identically processed version.

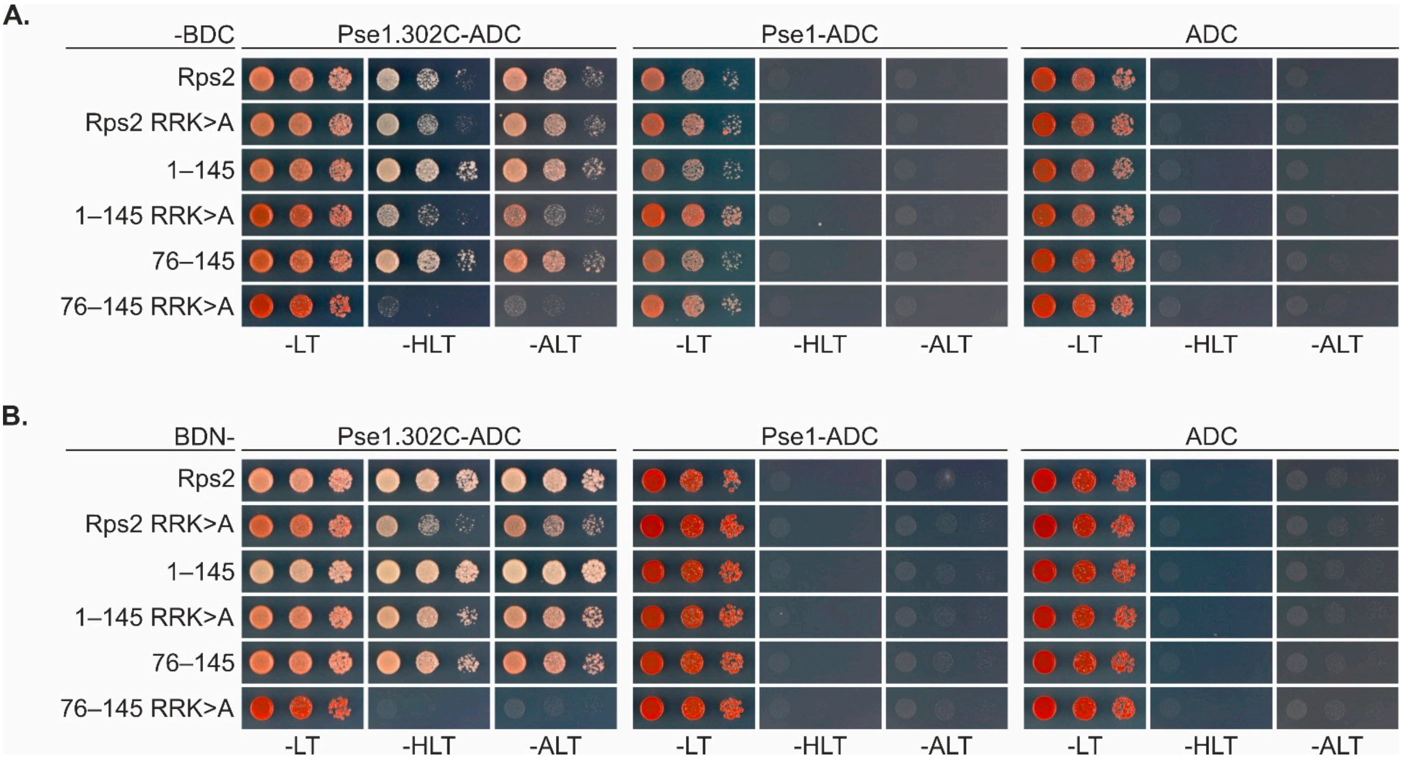

Supplementary Figure S6. **Yeast two-hybrid (Y2H) interaction of Rps2 and Pse1.** Pse1 lacking the 301 N-terminal amino acids (Pse1.302C), full-length Pse1, both C-terminally fused to the Gal4 activation domain (AD), and the Gal4 activation domain alone (negative control) were tested for interaction with Rps2 and the indicated fragments thereof (including when indicated the RRK>A exchanges) containing the Gal4 DNA-binding domain (BD) either at the C-terminal (**A.**) or the N-terminal end (**B.**). Growth on SDC-his-leu-trp plates (labeled -HLT) indicates a weak interaction; growth on SDC-ade-leu-trp plates (labeled -ALT) indicates a strong Y2H interaction. SDC-leu-trp (labeled -LT) served as growth control.

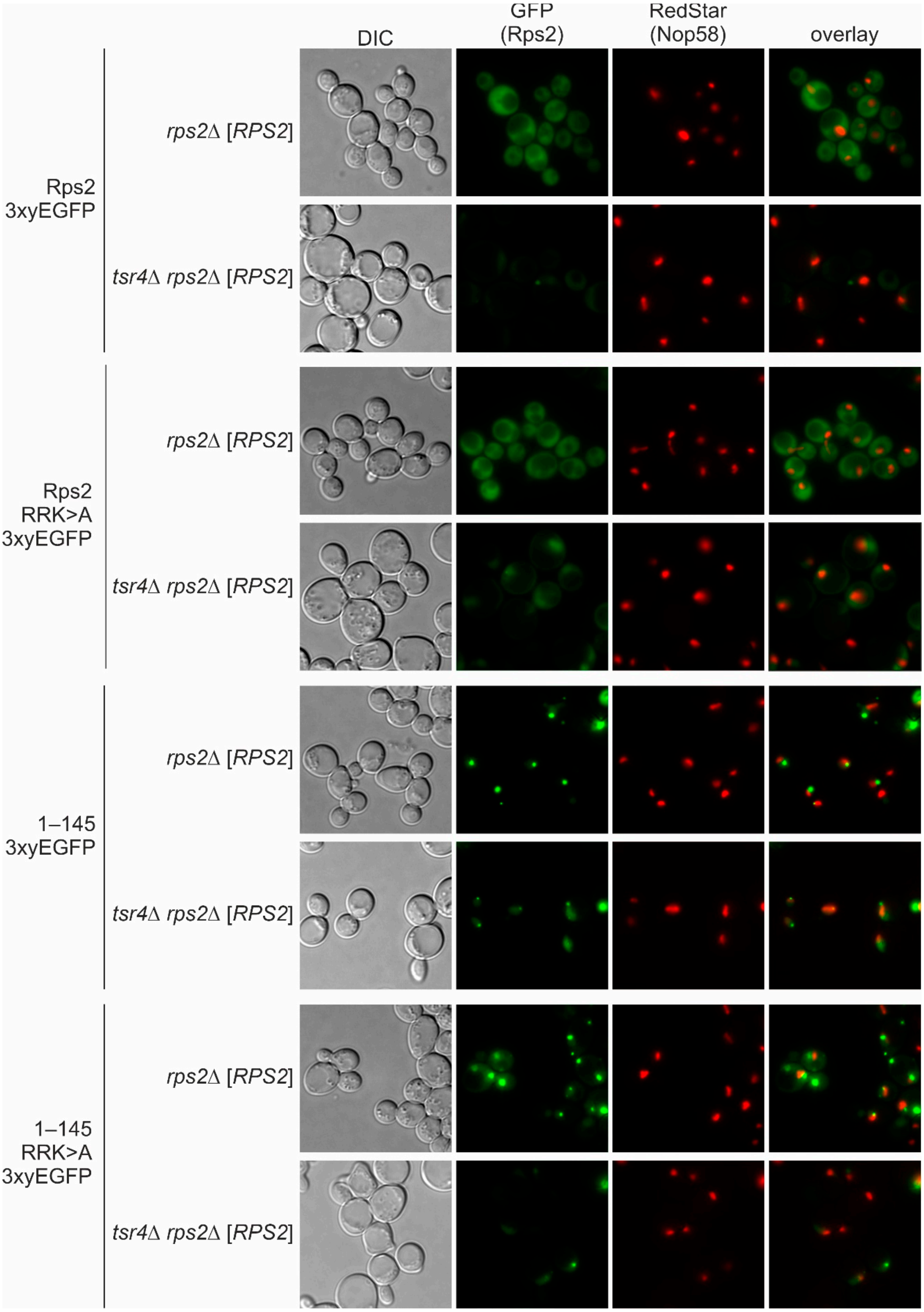

Supplementary Figure S7. Localization of Rps2-3xyEGFP reporter constructs in the absence of Tsr4. The same panels as in Figure 5 are shown, but with identical processing of all images.

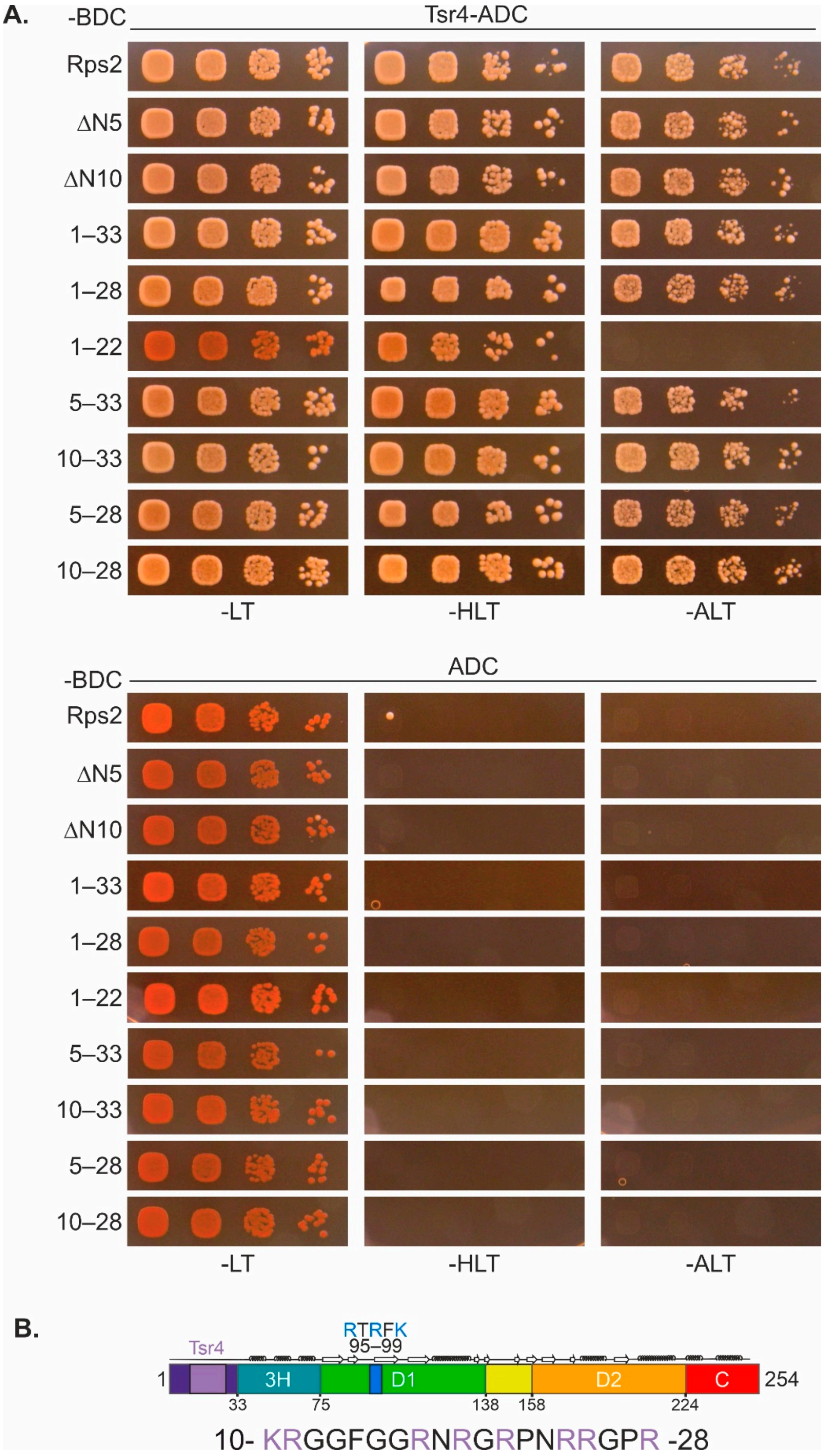

Supplementary Figure S8. **Mapping of the Tsr4-binding region on Rps2. A.** Yeast two-hybrid (Y2H) interaction assay between Tsr4, C-terminally fused to the Gal4 activation domain (AD), and Rps2 and fragments thereof, C-terminally fused to the Gal4 DNA-binding domain (BD), showing the same results as in Figure 6A but with additional constructs and negative controls (Rps2-BDC constructs against the non-fused AD). **B.** Schematic representation of Rps2 highlighting the updated Tsr4-binding region. The amino acid sequence of the minimal Tsr4-binding region is depicted below, with basic amino acids indicated in violet.

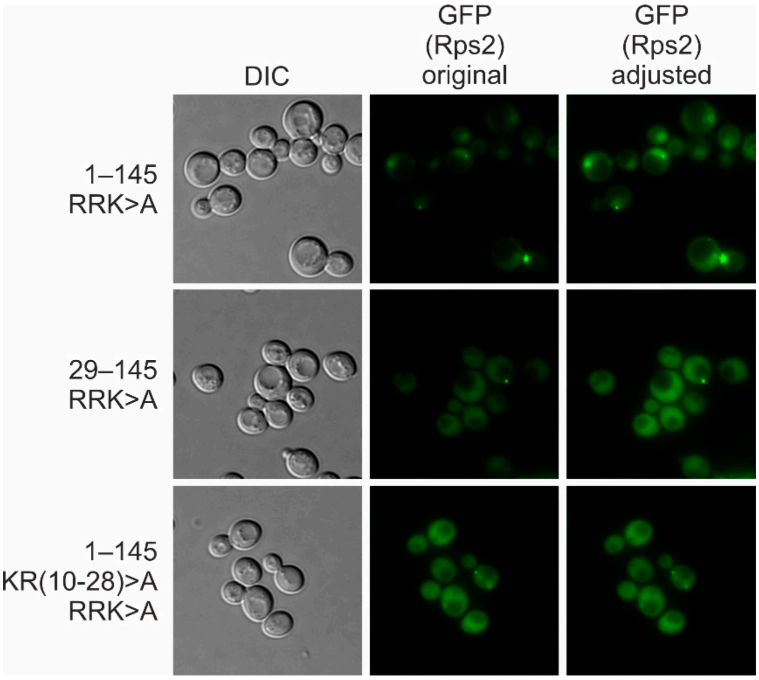

Supplementary Figure S9. **Localization of Rps2-3xyEGFP variants containing the $R_{95}R_{97}K_{99}$>A exchanges.** The images for which the intensities were adjusted in Figure 6B are shown in the original, identically processed version.

Supplementary Table S1. Yeast strains

name	genotype	source
W303	*MAT***a**/*MATα ade2-1/ade2-1 his3-11,15/his3-11,15 leu2-3,112/leu2-3,112 trp1-1/trp1-1 ura3-1/ura3-1 can1-100/can1-100*	[45]
C303	*MAT***a** *ADE2 his3-11,15 leu2-3,112 trp1-1 ura3-1 can1-100*	[15]
YDK11-5A	[W303] *MATα ade3*Δ::kanMX4	[46]
NOP58-yEmCherry	[W303] *MATα NOP58*-yEmCherry::natNT2 *ade3*Δ::kanMX4	[18]
PJ69-4A	*MAT***a** *trp1-901 leu2-3,112 ura3-52 his3-200 gal4Δ gal80Δ LYS2*::*GAL1-HIS3 GAL2-ADE2 met2*::*GAL7*-lacZ	[47]
srp1-31	*MAT***a** *ade2 his3 leu2 trp1 ura3 can1-100 srp1-31*	[48]
kap95 ts	*MAT***a** *his3 leu2 trp1 ura3 can1-100 kap95-ts*	Ed Hurt lab, backcross of PSY1103 [49] with W303
KAP104 shuffle	[W303] *MATα kap104*Δ::natNT2 *ade3Δ::kanMX4* pRS316-*KAP104 (URA3)*	[18]
pse1-1	*MAT***a** *his3 leu2 trp1 ura3 can1-100 pse1-1*	[23]
kap123Δ	*MATα his3 leu2 trp1 ura3 can1-100 kap123Δ::HIS3*	[23]
NOP58-RedStar2 *RPS2* shuffle	[C303] *MAT***a** *NOP58*-RedStar2::natNT2 *rps2*Δ::kanMX4 [pRS316-*RPS2*]	[7]
NOP58-RedStar2 *RPS2* shuffle *tsr4Δ*	[C303] *MAT***a** *NOP58*-RedStar2::natNT2 *rps2*Δ::kanMX4 *tsr4*Δ::*HIS3*MX4 [pRS316-*RPS2*]	[7]
RPS2 shuffle	[C303] *MATα rps2*Δ::kanMX4 [pRS316-*RPS2*]	This study
RPS2 shuffle *tsr4Δ*	[C303] *MAT***a** *rps2*Δ::kanMX4 *tsr4*Δ::*HIS3*MX4 [pRS316-*RPS2*]	This study

Supplementary Table S2. ***S. cerevisiae*** **plasmids.**

name	relevant information	source
pADH111-*RPS2*-$(GA)_5$-3xyEGFP	CEN, *LEU2*, P*ADH1*, T*ADH1*, C-terminal $(GA)_5$-3xyEGFP	[7]
pADH111-*RPS2(1–42)*-$(GA)_5$-3xyEGFP	CEN, *LEU2*, P*ADH1*, T*ADH1*, C-terminal $(GA)_5$-3xyEGFP	this study
pADH111-*RPS2(23–75)*-$(GA)_5$-3xyEGFP	CEN, *LEU2*, P*ADH1*, T*ADH1*, C-terminal $(GA)_5$-3xyEGFP	this study
pADH111-*RPS2(76–145)*-$(GA)_5$-3xyEGFP	CEN, *LEU2*, P*ADH1*, T*ADH1*, C-terminal $(GA)_5$-3xyEGFP	this study
pADH111-*RPS2(118–218)*-$(GA)_5$-3xyEGFP	CEN, *LEU2*, P*ADH1*, T*ADH1*, C-terminal $(GA)_5$-3xyEGFP	this study
pADH111-*RPS2(175–254)*-$(GA)_5$-3xyEGFP	CEN, *LEU2*, P*ADH1*, T*ADH1*, C-terminal $(GA)_5$-3xyEGFP	this study
pADH111-*rps2(76–145)*.$R_{95}R_{97}K_{99}$>A-$(GA)_5$-3xyEGFP	CEN, *LEU2*, P*ADH1*, T*ADH1*, C-terminal $(GA)_5$-3xyEGFP, $R_{95}R_{97}K_{99}$>A exchanges	this study
pADH111-*RPS2(1–145)*-$(GA)_5$-3xyEGFP	CEN, *LEU2*, P*ADH1*, T*ADH1*, C-terminal $(GA)_5$-3xyEGFP	this study

pADH111-*rps2(1–145)*.$R_{95}R_{97}K_{99}$>A-(GA)$_5$-3xyEGFP	CEN, *LEU2*, P*ADH1*, T*ADH1*, C-terminal (GA)$_5$-3xyEGFP, $R_{95}R_{97}K_{99}$>A exchanges	this study
pADH111-*RPS2(29–145)*-(GA)$_5$-3xyEGFP	CEN, *LEU2*, P*ADH1*, T*ADH1*, C-terminal (GA)$_5$-3xyEGFP	this study
pADH111-*rps2(29–145)*.$R_{95}R_{97}K_{99}$>A-(GA)$_5$-3xyEGFP	CEN, *LEU2*, P*ADH1*, T*ADH1*, C-terminal (GA)$_5$-3xyEGFP, $R_{95}R_{97}K_{99}$>A exchanges	this study
pADH111-*rps2(1–145)*.$KR_{10\text{-}28}$>A-(GA)$_5$-3xyEGFP	CEN, *LEU2*, P*ADH1*, T*ADH1*, C-terminal (GA)$_5$-3xyEGFP $KR_{10\text{-}28}$>A exchanges	this study
pADH111-*rps2(1–145)*-$KR_{10\text{-}28}$>A-$R_{95}R_{97}K_{99}$>A-(GA)$_5$-3xyEGFP	CEN, *LEU2*, P*ADH1*, T*ADH1*, C-terminal (GA)$_5$-3xyEGFP, $KR_{10\text{-}28}$>A and $R_{95}R_{97}K_{99}$>A exchanges	this study
pADH111-*RPS2(76–145)*-TAP	CEN, *LEU2*, P*ADH1*, T*ADH1*, C-terminal TAP-tag	this study
pADH111-*rps2(76–145)*.$R_{95}R_{97}K_{99}$>A-TAP	CEN, *LEU2*, P*ADH1*, T*ADH1*, C-terminal TAP-tag, $R_{95}R_{97}K_{99}$>A exchanges	this study
pADH111-TAP	CEN, *LEU2*, P*ADH1*, T*ADH1*, TAP-tag	this study
pCUP111-yEGFP-(GA)$_5$-TurboID-2xHA	CEN, *LEU2*, P*CUP1*, T*ADH1*, C-terminal (GA)$_5$-TurboID-2xHA	this study
pCUP111-SV40NLS-yEGFP-(GA)$_5$-TurboID-2xHA	CEN, *LEU2*, P*CUP1*, T*ADH1*, C-terminal (GA)$_5$-TurboID-2xHA	[43]
pCUP111-*RPS2*-(GA)$_5$-TurboID-2xHA	CEN, *LEU2*, P*CUP1*, T*ADH1*, C-terminal (GA)$_5$-TurboID-2xHA	this study
pCUP111-*rps2*.$R_{95}R_{97}K_{99}$>A-(GA)$_5$-TurboID-2xHA	CEN, *LEU2*, P*CUP1*, T*ADH1*, C-terminal (GA)$_5$-TurboID-2xHA, $R_{95}R_{97}K_{99}$>A exchanges	this study
pCUP111-*RPS2(1–145)*-(GA)$_5$-TurboID-2xHA	CEN, *LEU2*, P*CUP1*, T*ADH1*, C-terminal (GA)$_5$-TurboID-2xHA	this study
pCUP111-*rps2(1–145)*.$R_{95}R_{97}K_{99}$>A-(GA)$_5$-TurboID-2xHA	CEN, *LEU2*, P*CUP1*, T*ADH1*, C-terminal (GA)$_5$-TurboID-2xHA, $R_{95}R_{97}K_{99}$>A exchanges	this study
pCUP111-*RPS2(76–145)*-(GA)$_5$-TurboID-2xHA	CEN, *LEU2*, P*CUP1*, T*ADH1*, C-terminal (GA)$_5$-TurboID-2xHA	this study
pCUP111-*rps2(76–145)*.$R_{95}R_{97}K_{99}$>A-(GA)$_5$-TurboID-2xHA	CEN, *LEU2*, P*CUP1*, T*ADH1*, C-terminal (GA)$_5$-TurboID-2xHA, $R_{95}R_{97}K_{99}$>A exchanges	this study
pGAG4ADC181	2μ, *LEU2*, P*ADH1*, T*ADH1*, C-terminal (GA)$_5$-G4AD	[16]
pGAG4ADC181-*PSE1*	2μ, *LEU2*, P*ADH1*, T*ADH1*, C-terminal (GA)$_5$-G4AD	this study
pGAG4ADC181-*PSE1.302C*	2μ, *LEU2*, P*ADH1*, T*ADH1*, C-terminal (GA)$_5$-G4AD	this study
pG4BDN112-*RPS2*	2μ, *TRP1*, P*ADH1*, T*ADH1*, N-terminal G4BD	this study
pG4BDN112-*rps2*.$R_{95}R_{97}K_{99}$>A	2μ, *TRP1*, P*ADH1*, T*ADH1*, N-terminal G4BD, $R_{95}R_{97}K_{99}$>A exchanges	this study
pG4BDN112-*RPS2(1–145)*	2μ, *TRP1*, P*ADH1*, T*ADH1*, N-terminal G4BD	this study
pG4BDN112-*rps2(1–145)*.$R_{95}R_{97}K_{99}$>A	2μ, *TRP1*, P*ADH1*, T*ADH1*, N-terminal G4BD, $R_{95}R_{97}K_{99}$>A exchanges	this study
pG4BDN112-*RPS2(76–145)*	2μ, *TRP1*, P*ADH1*, T*ADH1*, N-terminal G4BD	this study
pG4BDN112-*rps2(76–145)*.$R_{95}R_{97}K_{99}$>A	2μ, *TRP1*, P*ADH1*, T*ADH1*, N-terminal G4BD, $R_{95}R_{97}K_{99}$>A exchanges	this study
pGAG4BDC112-*RPS2*	2μ, *TRP1*, P*ADH1*, T*ADH1*, C-terminal (GA)$_5$-G4BD	this study
pGAG4BDC112-*rps2*.$R_{95}R_{97}K_{99}$>A	2μ, *TRP1*, P*ADH1*, T*ADH1*, C-terminal (GA)$_5$-G4BD, $R_{95}R_{97}K_{99}$>A exchanges	this study
pGAG4BDC112-*RPS2(1–145)*	2μ, *TRP1*, P*ADH1*, T*ADH1*, C-terminal (GA)$_5$-G4BD	this study
pGAG4BDC112-*rps2(1–145)*.$R_{95}R_{97}K_{99}$>A	2μ, *TRP1*, P*ADH1*, T*ADH1*, C-terminal (GA)$_5$-G4BD, $R_{95}R_{97}K_{99}$>A exchanges	this study

pGAG4BDC112-*RPS2(76–145)*	2µ, *TRP1*, P*ADH1*, T*ADH1*, C-terminal $(GA)_5$-G4BD	this study
pGAG4BDC112-*rps2(76–145)*.$R_{95}R_{97}K_{99}$>A	2µ, *TRP1*, P*ADH1*, T*ADH1*, C-terminal $(GA)_5$-G4BD, $R_{95}R_{97}K_{99}$>A exchanges	this study
pG4ADC111-*TSR4*	CEN, *LEU2*, P*ADH1*, T*ADH1*, C-terminal G4AD	[7]
pG4BDC22-*RPS2*	CEN, *TRP1*, P*ADH1*, T*ADH1*, C-terminal G4BD	[7]
pGAG4BDC22-*RPS2*ΔN5	CEN, *TRP1*, P*ADH1*, T*ADH1*, C-terminal $(GA)_5$-G4BD	this study
pGAG4BDC22-*RPS2*ΔN10	CEN, *TRP1*, P*ADH1*, T*ADH1*, C-terminal $(GA)_5$-G4BD	this study
pGAG4BDC22-*RPS2(1–33)*	CEN, *TRP1*, P*ADH1*, T*ADH1*, C-terminal $(GA)_5$-G4BD	this study
pGAG4BDC22-*RPS2(1–28)*	CEN, *TRP1*, P*ADH1*, T*ADH1*, C-terminal $(GA)_5$-G4BD	this study
pG4BDC22-*RPS2(1–22)*	CEN, *TRP1*, P*ADH1*, T*ADH1*, C-terminal $(GA)_5$-G4BD	[7]
pGAG4BDC22-*RPS2(5–33)*	CEN, *TRP1*, P*ADH1*, T*ADH1*, C-terminal $(GA)_5$-G4BD	this study
pGAG4BDC22-*RPS2(10–33)*	CEN, *TRP1*, P*ADH1*, T*ADH1*, C-terminal $(GA)_5$-G4BD	this study
pGAG4BDC22-*RPS2(5–28)*	CEN, *TRP1*, P*ADH1*, T*ADH1*, C-terminal $(GA)_5$-G4BD	this study
pGAG4BDC22-*RPS2(10–28)*	CEN, *TRP1*, P*ADH1*, T*ADH1*, C-terminal $(GA)_5$-G4BD	this study
pRS314	CEN, *TRP1*	[50]
pRS316	CEN, *TRP1*	[50]
YCplac22-*KAP104*	CEN, *TRP1*, P*KAP104*, T*KAP104*	[17]
pRS314-*kap104-16*	CEN, *TRP1*, P*KAP104*, T*KAP104*	[51]
pRS314-*KAP123*	CEN, *TRP1*, P*KAP123*, T*KAP123*	this study

Supplementary Table S3: TurboID proximity labeling data

The table is provided as an Excel Sheet.
